# Effects of Yoga on Blood Glucose and Lipid Profile of Type 2 Diabetes Patients Without Complications: A Systematic Review and Meta-Analysis

**DOI:** 10.3389/fspor.2022.900815

**Published:** 2022-06-23

**Authors:** Shanshan Chen, Shilin Deng, Yang Liu, Tiantian Yin

**Affiliations:** School of Physical Education, Wuhan University of Technology, Wuhan, China

**Keywords:** meta-analysis, type 2 diabetes, yoga, blood glucose, lipid profile

## Abstract

**Background:**

Type II diabetes mellitus (T2DM) has become a worldwide public health problem. Although it has been empirically established that physical activity is a promising therapeutical approach to the prevention and management of T2DM, the effectiveness of yoga on T2DM has not yet reached an agreement across studies and also needs an updated synthetic examination.

**Purpose:**

The purpose of this study was to examine the effect of yoga training on diabetes-related indicators compared with usual care.

**Methods:**

The review protocol of this study has been registered in the PROSPERO with a registration number CRD42021267868. A systematic literature search through electronic databases was conducted to identify yoga-based intervention (i.e., randomized controlled trial [RCT]; e.g., yogic postures, movements, breathing, and meditation) studies reporting outcomes on glycosylated hemoglobin (HbA1c), fasting blood glucose (FBG), postprandial blood glucose (PPBG), total cholesterol (TC), triglycerides (TG), and body mass index (BMI). A number of two researchers manually reviewed and assessed each article using the Cochrane Risk of Bias Tool 2.0. The literature search identified 296 eligible entries, of which 13 were finalized after screening using predefined inclusion and exclusion criteria. The extracted data (group mean and standard deviation at posttest) were synthesized using random-effects meta-analyses. Finally, potential moderators were explored using subgroup analysis and sensitivity analysis.

**Results:**

The standardized mean difference for the effects of yoga was significant on HbA1c (MD = −0.47; 95%CI: −0.77, −0.16; *Z* = 3.02, *p* = 0.003), FBG (SMD = −0.92; 95%CI: −1.55, −0.29; *Z* = 2.87, *p* = 0.004), PPBG (SMD = −0.53; 95%CI: −0.86, −0.21; *Z* = 3.20, *p* = 0.001), and TG (SMD = −0.32; 95%CI: −0.54, −0.10; *Z* = 2.86, *p* = 0.004). However, yoga effect was not observed on TC (SMD = −0.84; 95%CI: −1.71, 0.04; *Z* = 1.87, *p* = 0.06) and BMI (MD = −0.63; 95%CI: −1.42, 0.16; *Z* = 1.57, *p* = 0.12).

**Conclusion:**

The findings suggest that yoga can improve the biochemical indices of blood glucose and the lipid profile of patients with T2DM. Therefore, yoga can be prescribed as an effective and active complementary treatment for T2DM. However, this study only tested yoga as a short-term treatment. In the future, rigorous RCTs with a larger sample size may be carried out to examine the long-term effect of yoga on T2DM.

**Systematic Review Registration:**

https://www.crd.york.ac.uk/PROSPERO/display_record.php?RecordID=267868, identifier: CRD42021267868.

## Introduction

Type 2 diabetes mellitus (T2DM) has become a serious public health problem across the globe, which is typically characterized by impaired insulin secretion and insulin resistance, and seriously affects the quality of life of patients. Individuals with T2DM were subjected to many life-threatening health problems, resulting in higher medical care costs, faded quality of life, and a higher risk of mortality (Baena-Díez et al., 2016). According to the International Diabetes Federation, the prevalence of diabetes worldwide is now estimated to be over 10%, and the cases of diabetes were dominated by T2DM. By 2045, the absolute number of patients with T2DM will increase by 46% (Sun et al., 2022). Obviously, T2DM has become a global epidemic. Apart from uncontrolled factors, physical inactivity, unhealthy diet intake, overweight, and obesity were thought to be the main contributors to diabetes (Hu, 2011). Regardless of the type of diabetes, patients were required to control their blood glucose through receiving medication, exercise prescriptions, and special dietary plans. However, therapies incorporating exercise were believed to be one of the safe and healthy approaches for T2DM treatment. Currently, there are burgeoning unconventional auxiliary treatment options available for patients with diabetes for better control of their blood glucose levels such as yoga, massage therapy, and acupuncture (Pandey et al., 2011). Ascertaining the effectiveness of yoga on T2DM through empirical examination is therefore warranted. To date, yoga as adjuvant therapy for diabetes has not yet been thoroughly investigated for agreement across studies, especially for patients with T2DM without severe metabolic syndrome (Dutta et al., 2021).

According to the American Diabetes Association, moderate-intensity aerobic exercise plays a pivotal role in managing diabetes-induced metabolic disorders (American Diabetes Association, 2019). In fact, insufficient physical activity leads to an increased risk of obesity (Kim et al., 2017), which is a possible reason for the occurrence of T2DM and also a considerable predictor of mortality and complications in patients with T2DM (American College of Sports Medicine, 2013). Therefore, one of the key points of T2DM care is to provide a tailored physical activity recommendation while taking into account patients' complex health conditions (Lin et al., 2012). As a mind-and-body integrated exercise, yoga capitalizes on the capability of meditation to enhance physical health, which appears particularly suitable for the fitness condition of patients with diabetes. Yoga is underpinned by one of the six philosophical systems in ancient India advocating the ideal way of lifestyle. Meanwhile, yoga can be of moderate exercise intensity under specific types of training (Larson-Meyer, 2016). In recent years, yoga has been much more fashionable across the globe, and the use of yoga as a therapy is developing rapidly (Jeter et al., 2015). Practically, yoga has played a significant role in the treatment and prevention of diabetes. Several experimental studies examining the effectiveness of yoga on T2DM have shown favorable results in blood glucose, lipid profile, oxidative stress, blood pressure, anthropometric indicators, and quality of life (Gordon et al., 2008; Hegde et al., 2011, 2020; Shantakumari and Sequeira, 2013; Datey et al., 2017; Balaji et al., 2020; Sharma et al., 2020; Nair et al., 2021; Ranga et al., 2021; Sivapuram et al., 2021). Additionally, favorable results were also observed in mental health, functional capacity, wellbeing, sleep quality, and body composition among patients with diabetes, as a result of receiving yoga practice (Akhtar et al., 2013; Innes and Selfe, 2016; Miles et al., 2016; Rshikesan et al., 2017).

Prior intervention studies examining the response of T2DM to yoga training showed typical characteristics across philosophical underpinning, exercise type, dosage, and population. The Hindu religious perspectives underlie the physical practices of yoga which also frames the ideology for yogic meditation (De Michelis, 2005). Most yogic practices adopted in the interventions focused primarily on postures (asanas), usually with the additions of breath control (pranayama), and/or sometimes also incorporating elements of concentration or meditation; however, this varies with different styles or schools of yoga (e.g., Hatha vs. integrated yoga) that are practiced in different areas of the world and/or for different purposes (De Michelis, 2005; Sengupta, 2012). The reported dosage of promising yoga interventions showed variations in the duration of each yoga session (45–90 min), frequency (2–3 times/week to every day), and total length of intervention (40 days−6 months). The reported age of subjects ranged from 30 to 83.7 years. It is worth noting that most of the study subjects are adults from India, which may be due to the origin of yoga in India (Jeter et al., 2015).

Type 2 diabetes mellitus is typified by hyperglycemia in the presence of insulin resistance (American Diabetes Association, 2014). Therefore, keeping glucose levels within a healthy range is the most important recommendation for diabetes management (Inzucchi et al., 2012). Other key-related hemodynamic and metabolic abnormalities characterizing T2DM include elevated blood pressure, dyslipidemia, and increased oxidative stress (Wellen and Hotamisligil, 2005; Innes and Vincent, 2007; Rana et al., 2007). It is known that people who are overweight or obese have a higher risk of developing T2DM. Body mass index (BMI) is a measurement of body weight status. A meta-analysis with prospective cohort studies reported that overweight (BMI: 25–30 kg/m^2^) or obese (BMI: >30 kg/m^2^) individuals were more likely to have T2DM (Mi et al., 2020). It can be seen that BMI plays an important role in diabetes management. Considering the above reasons, blood glucose, lipid profile, and BMI were considered the variables of interest in this study. Furthermore, the risk for T2DM increases with age, and even individuals as early as childhood are also subjected to such disease (Yoon et al., 2006). Among those under 60 years old, 41.1% of deaths are diabetes-related (IDF Diabetes Atlas, 2015). Given that, T2DM as a life-threatening risk factor can occur at any time point across the entire lifespan, which deserves more epidemiological and medical investigation for a healthier way of prevention and/or treatment.

Despite the studies of systematic review and meta-analysis having been published previously (Ramamoorthi et al., 2019; Wibowo et al., 2021), the role of yoga in diabetes treatment, especially whether yoga can significantly improve blood glucose and lipid profile, was still under debate. Besides, most of the prior meta-analyses had included prediabetes or T2DM patients with other symptoms such as severe metabolic syndrome rather than merely patients with T2DM alone and rarely confirmed whether the patients have complications, making it difficult to generalize the results to other settings. In addition, the inclusion criteria of prior studies were problematic in that the inclusion of low-dose (i.e., intervening length ≤4 weeks) studies would jeopardize the credibility of the results. Given the above factors, this study was an update as follows. First, we strictly restricted our analyses to subjects with T2DM. This study only evaluated the effect of yoga on patients with uncomplicated T2DM, not taking into account patients with complications, prediabetes, gestational diabetes, etc., and further explored the effect of yoga on patients of different ages. Second, we rigorously developed inclusion criteria based on the Cochrane manual and included only randomized controlled trials, excluding case–control or quasi-experimental studies, and low-dose studies. Finally, in addition to observing the effects of yoga on blood glucose and lipid profiles, we also assessed the effect of yoga on BMI, which is necessary because most patients with T2DM are with abnormal BMI. Not only that, we expanded the search scope and refreshed the search years. Therefore, the purpose of this study was to evaluate available evidence from existing randomized controlled trials concerning the effect of yoga-based intervention (e.g., yogic postures, movements, breathing, and meditation) on the biochemical indicators of blood glucose, lipid profile, and BMI in patients with T2DM and expected to provide an evidence-based reference for the treatment of diabetes with yoga practice.

## Methods

The review protocol of this study has been registered in the PROSPERO and assigned the registration number CRD42021267868. All review procedures followed the Preferred Reporting Items for Systematic Reviews and Meta-Analyses (PRISMA) recommendation (Moher et al., 2009). The PRISMA checklist can be found in the Supplemental Materials.

### Identification of Studies

The Cochrane handbook provided the guideline for conducting and reporting this systematic review and meta-analysis study (Higgins, 2011). A systematic search was conducted to investigate the yoga effect on T2DM. After identifying relevant Medical Subject Headings (MeSH), six electronic databases were searched by the first author including Cochrane Library, Medline, EMBASE, PubMed, Web of Science, and FMRS from their inception through July 2021. The keywords used (in possible combinations and variations) in the search were built as follows: (yoga [MeSH] OR exercise [MeSH] OR physical activity [MeSH]) AND (type 2 diabetes mellitus [MeSH]) AND (fasting blood glucose [MeSH] OR fasting blood sugar [MeSH] OR fasting plasma glucose [MeSH]) AND (postprandial blood sugar [MeSH] OR postprandial blood glucose [MeSH]) AND (total cholesterol [MeSH]) AND (Triglycerides [MeSH]) AND (glycated hemoglobin [MeSH] OR glycosylated hemoglobin [MeSH] OR HbA1c [tiab]) AND (body mass index OR BMI [tiab]) AND (randomized controlled trial [MeSH] OR RCT [tiab]). Titles and abstracts of the citations were scanned to identify potential articles. Potentially eligible articles were retrieved for more detailed review. Eligible trials were limited to adult human subjects, and only trials published with the full text and written in English were included in this study.

### Criteria for Inclusion and Exclusion

The following eligibility criteria were applied for screening: (1) setting and population: adult patients with T2DM (as diagnosed by a clinician, or using recognized diagnostic criteria) with confirmed disease regardless of gender; (2) study design: randomized controlled trials; (3) intervention: yoga-based intervention (e.g., yogic postures, movements, breathing, and meditation) / program; (4) comparison: the control group only receives usual care or alternate program; (5) geographic origin: countries across the globe; (6) language: English; (7) age: <70 years old; (8) study duration: 10–24 weeks. Studies violating one of the following criteria were excluded: (1) suspected patients with T2DM without further confirmatory testing; (2) literature review; (3) with no control group; (4) without sufficient data/statistics for referential analysis and/or without full text; and (5) patients with gestational diabetes mellitus. The electronic database search was complemented with a manual search to identify any missing studies from the reference lists of all included articles. A number of two research assistants (C and D) independently screened each article and any discrepancies in eligibility were discussed before a decision was made for inclusion or exclusion.

### Risk of Bias Assessment

The risk of bias was evaluated using the Cochrane Risk of Bias Tool (Higgins, 2011) by two research assistants (C and D) independently, which rated each article against six criteria: (1) selection bias: whether details of random sequence generation and allocation concealment were sufficiently described; (2) performance bias: whether blinding of participants and personnel as well as blinding efficacy was sufficiently described; (3) detection bias: whether blinding of outcome assessment was sufficiently described; (4) attrition bias, whether details and reasons of attrition and exclusion were sufficiently described; (5) reporting bias: whether concerns for possible selective outcomes reporting were clearly stated; (6) other bias: whether any other important concerns that were not addressed in the above five criteria were described. The assessment to each criterion has three levels: low risk of bias, unclear risk of bias, and high risk of bias. A third research assistant (L) would jump in to solve the problem whether there were discrepancies between the two research assistants.

### Data Extraction

The information and data extraction for the included articles were conducted by two research assistants (C and D) independently using a predefined data extraction form, including the first author name, year of publication, sample size, intervention duration, research design, demographic details (i.e., gender and age), and group mean and standard deviation (SD) at posttest. The extracted data and information from the included studies were entered and saved in an Excel spreadsheet using a purpose-built template. For RCTs with three-arms or more, only data from the yoga group and control group were used for comparison. Any disagreement between the two reviewers was discussed until a consensus was reached. But if disagreement was not resolved, a third research assistant (L) would sort out the conflict.

### Data Synthesis and Meta-Analysis

The literature review and meta-analysis were conducted using EndNote X8 and Review Manager (Revman) version 5.4.1, respectively, for bias processing, assessment, heterogeneity test, pooled data evaluation, bias graph, forest plot, and funnel plot. The meta-analysis only examined continuous outcome variables, and each mean difference was weighted according to the inverse variance method (weighted mean difference [MD]) (Higgins, 2011). When the same outcome was measured by different scales, the mean difference was standardized by dividing it by the within-group SD; the results were then weighted and the average is taken (standardized mean difference [SMD]). The MD or SMD in each study was pooled with a random-effects model (Higgins and Green, 2008). The *p*-value was set at 0.05 to be statistically significant. Within-group heterogeneity was evaluated using the *I*^2^ statistic (negligible: *p* > 0.10, heterogeneous: *p* ≤ 0.10), with low, moderate, and high heterogeneity levels set to be *I*^2^ values of 25, 50, and 75%, respectively (Higgins et al., 2003). For subgroup analyses, the heterogeneity between groups was also calculated using the *I*^2^ statistic.

### Subgroup and Sensitivity Analyses

Substantial differences were observed in session length, publication year, sample size, and age, across included studies, and thus, subgroup analysis was used. Subgroup analysis is one of the important methods to analyze the heterogeneous results or to answer questions about specific patients, intervention types, or research types. The subgroup moderator analysis was used to explain higher heterogeneity in this meta-analysis and examine whether the effects of yoga differed according to (1) session length, (2) age, and (3) sample size. To further investigate, sensitivity analysis was conducted for the meta-analyses by bringing out each study one by one from the meta-analysis and recomputing the effect size and *I*^2^ to evaluate the influence of each study on the newly emerged effect size.

### Publication Bias

A funnel plot was used for assessing the publication bias. A higher risk of publication bias depicts a much asymmetric distribution in the plot. The funnel plot is a commonly used method of identifying publication bias in meta-analysis. In the absence of bias, the dots in the plot should be clustered into an inverted funnel. If there is severe publication bias, the funnel plot looks asymmetrical and there is a blank in the bottom corner of the plot. In this case, the synthesized effect sizes calculated by meta-analyses may overestimate the overall efficacy of the included interventions (Higgins, 2011).

## Result

### Study Selection

**Figure 1:**
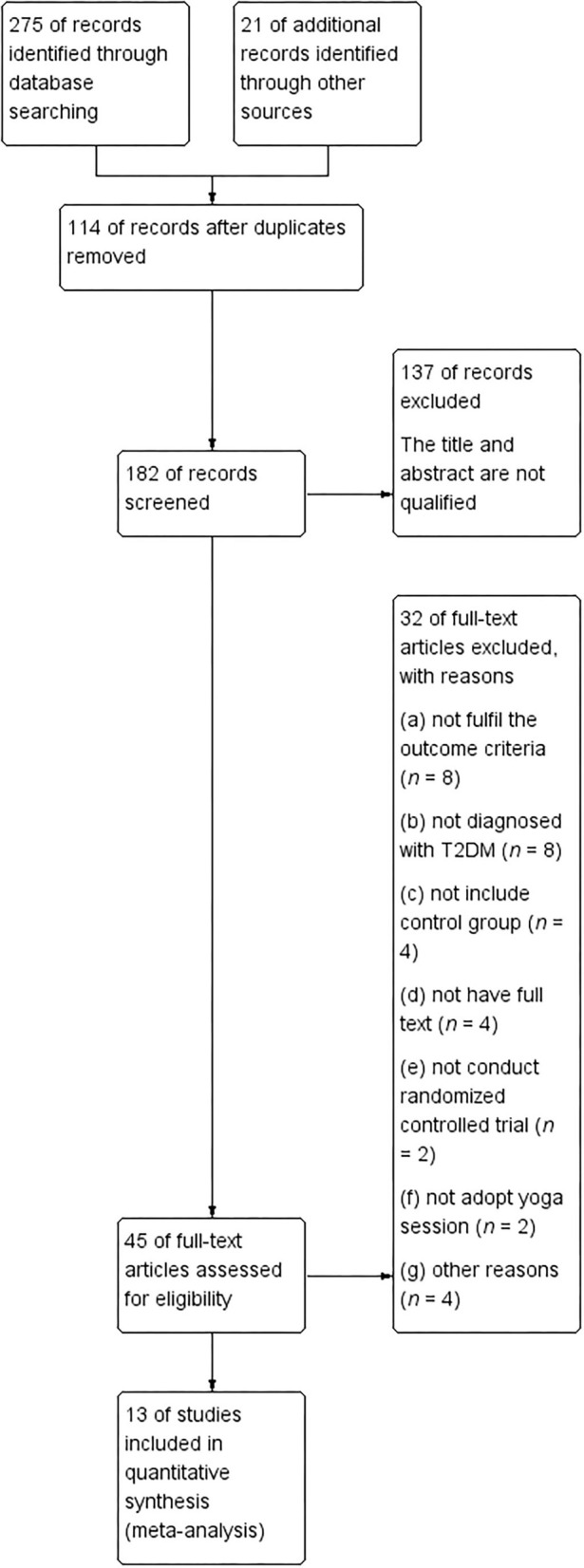
Flow chart for study inclusion and exclusion process.

Based on the participant, interventions, comparisons, and outcomes (PICO) principle (Higgins, 2011), the initial search retrieved 296 entries from six databases. After removing 114 duplicates, there were 182 studies left. We further excluded 137 articles with irrelevant titles and abstracts. After the first round of screening, 251 articles were excluded and 45 studies with full text received a further evaluation. In the second round of screening, 32 studies were excluded because of the following reasons: (a) not fulfill the outcome criteria (*n* = 8); (b) not diagnosed with T2DM (*n* = 8); (c) not include control group (*n* = 4); (d) not have full text (*n* = 4); (e) not conduct randomized controlled trial (*n* = 2); (f) not adopt yoga session (*n* = 2); and (g) other reasons (*n* = 4). Finally, only 13 RCT studies were included in the meta-analyses (Figure 1).

### Study Characteristics

Key characteristics of eligible studies are shown in Table 1. Overall, the total sample size was 1,335 (intervention = 672, control = 663). The sample sizes across all eligible studies ranged from 40 to 300. All participants were adults with T2DM (*M*_age_ = 53.2; *M*_*age*_ range: 41.3–63.8 years). A number of two studies did not specify patients' average age but were also included as an exception because the age range was provided meeting the inclusion criteria. The length of yoga intervention varied from 10 to 24 weeks with the majority of the studies receiving 12 weeks (62%) of intervention (Hegde et al., 2011, 2020; Shantakumari and Sequeira, 2013; Datey et al., 2017; Sreedevi et al., 2017; Ranga et al., 2021; Sivapuram et al., 2021; Viswanathan et al., 2021) and few studies using 24 weeks (two studies: 15%) (Gordon et al., 2008; Sharma et al., 2020), 16 weeks (two studies: 15%) (Balaji et al., 2020; Gupta et al., 2020), and 10 weeks (one study: 8%) (Nair et al., 2021). Each yoga session lasted for 45–120 min per day, and the frequency was 1–7 days per week. The majority of the studies did not explicitly state the yoga style used during the intervention.

**Table 1:** Characteristics of the included intervention studies.

**Study**	**N (female%)**	**Age mean (SD)**	**Group**	**Duration (min/d)**	**Frequency (d/wk)**	**Study length (wk)**	**Outcomes**
Balaji et al. (2020)	69 (32%)	49.6 (5.9)	YG, CT	60	3	16	Outcome measures included fasting blood sugar (FBS), postprandial blood sugar (PPBS), HbA1c, blood urea, and serum creatin.
Datey et al. (2017)	74 (0%)	41.3 (11.4)	AHJ+YG, YG, CT	60	7	12	Outcome measures included fasting blood sugar (FBS) postprandial blood sugar (PPBS) and the hemoglobin A1c (HbA1c).
Gordon et al. (2008)	154 (80.5%)	63.8	PT, YG, CT	120	1	24	Outcome measures included lipid profile, fasting blood glucose, concentration of MDA, PLA2 activity, and POX.
Gupta et al. (2020)	78 (44%)	50.6 (8.5)	YG, CT	45	3	16	Outcome measures included Hba1c, weight, body mass index, waist circumference, systolic blood pressure, diastolic blood pressure, FPG, total cholesterol, triglycerides, LDL cholesterol, and HDL cholesterol.
Hegde et al. (2011)	123	58.6 (9.4)	YG, CT	–	3	12	Outcome measures included BMI, waist circumference, waist-to-hip ratio, blood pressure, fasting plasma glucose (FPG), postprandial plasma glucose (PPPG), and Hba1c.
Hegde et al. (2020)	40 (50%)	57.3 (5.8)	YG, SYG	75–90	6	12	Outcome measures included MDA, GSH, vitamin C, SOD, fasting plasma glucose, glycosylated hemoglobin (Hba1c), waist circumference, body mass index (BMI), and blood pressure.
Nair et al. (2021)	45 (47%)	50.3 (4.3)	YG, CT	60	4	10	Outcome measures included fasting blood sugar (FBS), tail moment (TM), olive tail moment (OTM), total antioxidant capacity (TAC), low-density lipoprotein (LDL), high-density lipoprotein (HDL), triglycerides (TG), total cholesterol (TC), blood pressure, waist-to-hip ratio (WHR), and body mass index (BMI).
Ranga et al. (2021)	100	–	YG, CT	–	5	12	Outcome measures included blood pressure (BP), fasting blood glucose (FBG), postprandial blood glucose, and Hba1c.
Shantakumari and Sequeira (2013)	100 (49%)	45.0 (9.5)	YG, CT	60	7	12	Outcome measures included triglycerides (TG), total cholesterol (TC), HDL cholesterol, LDL cholesterol, and high-density lipoprotein e cholesterol (HDLeC).
Sharma et al. (2020)	104 (45%)	–	YG, CT	40	5	24	Outcome measures included glucose (fasting and postprandial) and lipid profile, body mass index (BMI), waist-to-hip ratio (WHR), TG, TC, HDL-c (enzymatic direct HDL method), low-density lipoprotein (LDL), very low-density lipoprotein (VLDL). AIP, The logarithm of molar ratio of TG to high-density lipoprotein cholesterol (TG/HDL cholesterol), was calculated.
Sivapuram et al. (2021)	81	56.7 (18.2)	YG, CT	60	7	12	Outcome measures included fasting blood sugar (FBS), postprandial blood sugar (PPBS), Hba1c, lipid profile, and BMI.
Sreedevi et al. (2017)	67 (100%)	51.9 (7.0)	PYG, YG, CT	60	2	12	Outcome measures included fasting plasma glucose, Hba1c, quality of life, Pharmacological adherence, BMI, waist-to-hip ratio (WHR), blood pressure, and total cholesterol.
Viswanathan et al. (2021)	300 (35%)	51.8 (7.7)	YG, CT	50	5	12	Outcome measures included anthropometric measurements, BMI, blood pressure, Pittsburgh sleep quality index (PSQI), glycated hemoglobin (HbA1c), TG, TC, HDL-C (enzymatic direct HDL method), low-density lipoprotein (LDL), very low-density lipoprotein (VLDL).

*Age was the average of all subjects in each study*.

*YG, yoga group; CT, control group; AHJ, Ayurveda herbal juices; PT, conventional physical training; SYG, sham yoga; PYG, peer sports yoga*.

### Quality and Risk of Bias Assessment

**Figure 2:**
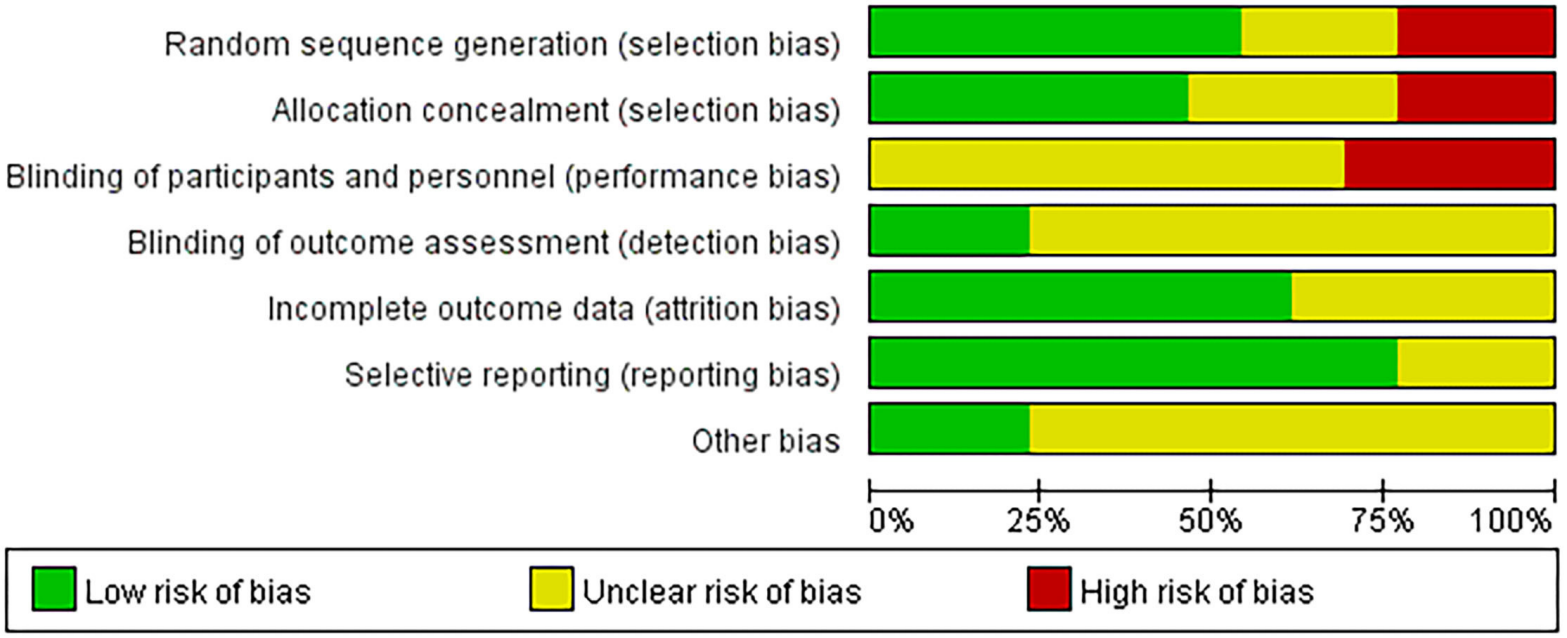
Risk of bias graph: judgment on each risk of bias item presented as a percentage across all included studies.

**Figure 3:**
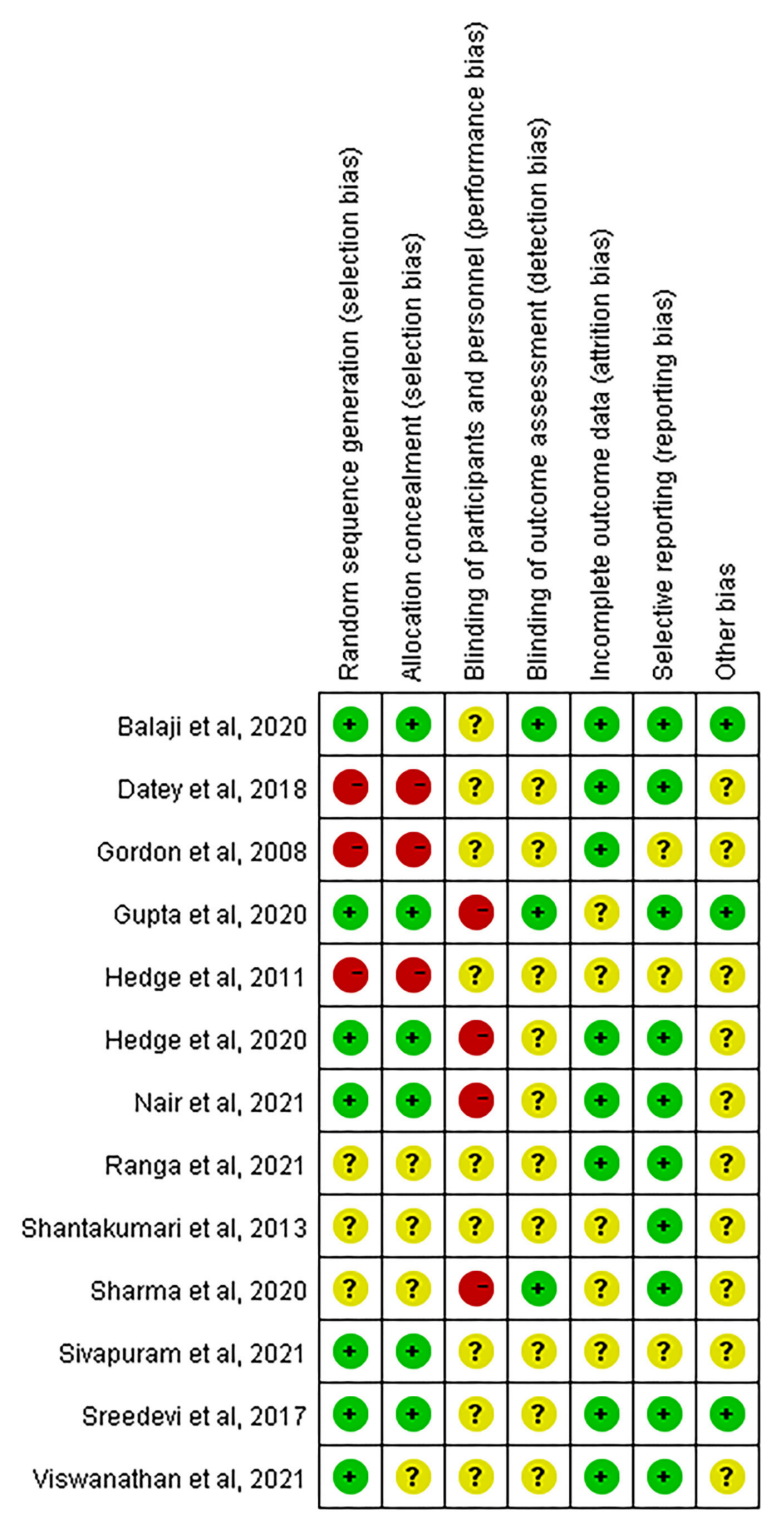
Risk of bias summary: judgment on each risk of bias item for each included study.

Quality and risk of bias assessment were conducted based on the Cochrane Risk of Bias Tool (Higgins, 2011). The summaries of the risk of bias across the 13 eligible studies are displayed in Figures 2, 3. The majority of studies (78%) were at low risk of bias for selective reporting. For random sequence generation, seven studies (54%) showed low risk, three studies (23%) were an unclear risk, and three studies (23%) have high risk. For allocation concealment, six studies (46%) adequately concealed allocation; three studies (23%) clearly stated that subjects and researchers were likely to predict distribution results, and four studies (31%) did not mention anything concerning allocation concealment. For binding of participants and personnel, all research was high risk and unclear risks. The rest of the risk biases was low risk and unclear risk (refer to Figures 2, 3 for details).

### Primary Outcomes

#### Effect of Yoga on HbA1c

**Figure 4:**
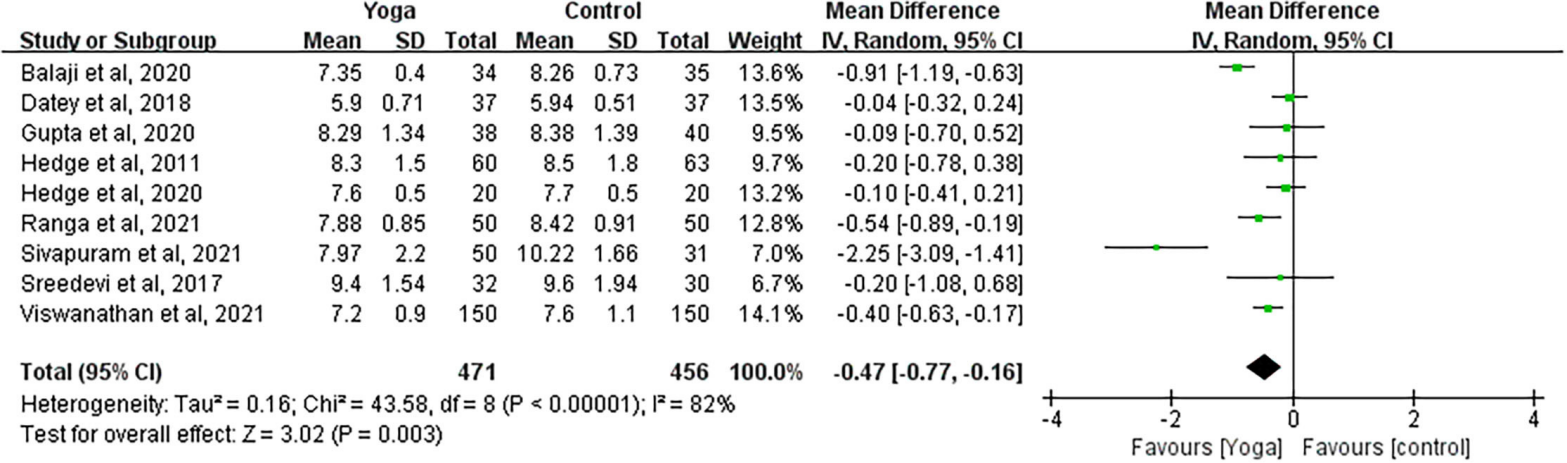
Forest plots for the effect of yoga on HbA1c.

Data involving 927 subjects with T2DM from nine eligible studies were analyzed to examine the effect of yoga on glycated hemoglobin (HbA1c). A number of four studies showed significant HbA1c decreases in the yoga group following the intervention compared to the control group (Figure 4). Results from the meta-analysis showed a significant overall mean difference favoring yoga group (MD = −0.47; 95%CI: −0.77, −0.16; *Z* = 3.02, *p* = 0.003). Heterogeneity was clearly significant for the pooled result of HbA1c (*df* = 8, *p* < 0.0001, *I*^2^ = 82%). We carried out subgroup analyses to investigate the potential sources of heterogeneity.

#### Effect of Yoga on FBG

**Figure 5:**
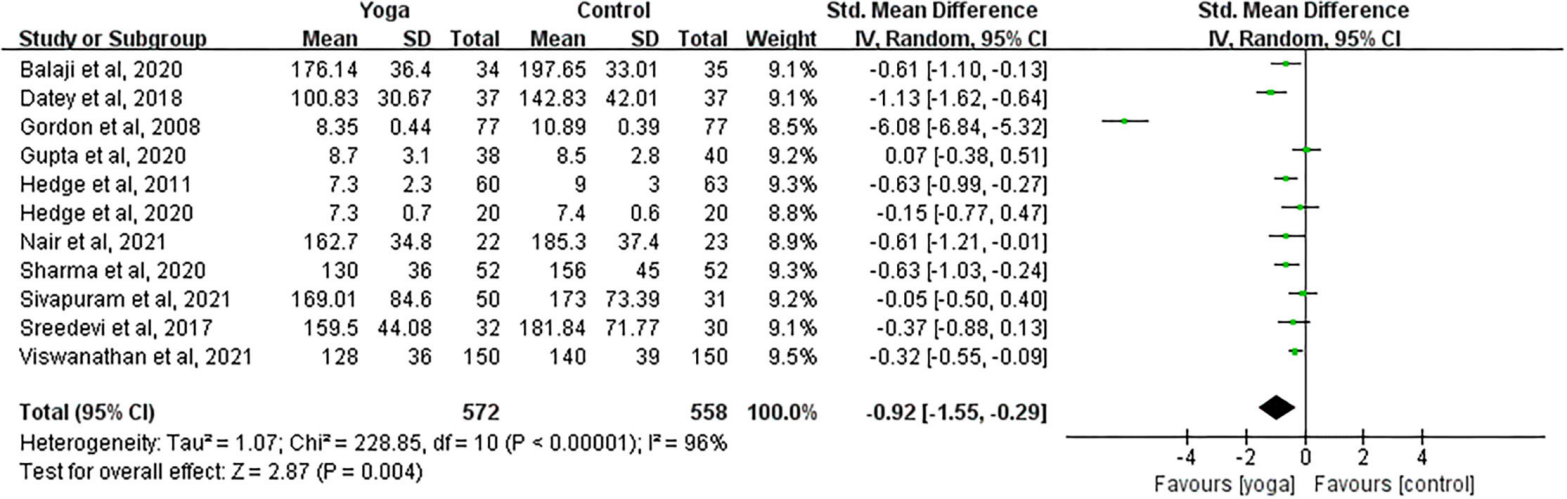
Forest plots of the effect of yoga on FBG.

Data involving 1,130 subjects with T2DM from 11 eligible studies were analyzed to ascertain the effect of yoga on fasting blood glucose (FBG). A total of seven studies showed significant FBG decreases in the yoga group following the intervention compared to the control group (Figure 5). Results from the meta-analysis showed a significant standardized mean difference favoring yoga group (SMD = −0.92; 95%CI: −1.55, −0.29; *Z* = 2.87, *p* = 0.004). Heterogeneity was clearly significant for the pooled result of FBG (*df* = 10, *p* < 0.0001, *I*^2^ = 96%). We carried out subgroup analyses to investigate the potential sources of heterogeneity.

#### Effect of Yoga on PPBG

**Figure 6:**
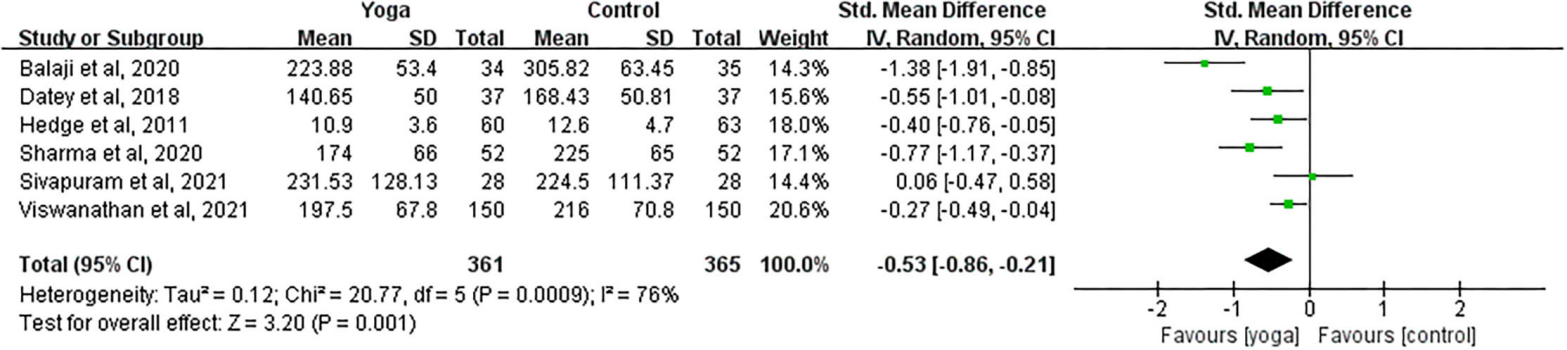
Forest plots of the effect of yoga on PPBG.

Data involving 726 subjects with T2DM from six eligible studies were analyzed to find out the effect of yoga on postprandial blood glucose (PPBG). The majority of the studies showed significant PPBG decreases in the yoga group following the intervention compared to the control group (Figure 6). Results from the meta-analysis showed a significant standardized mean difference favoring yoga group (SMD = −0.53; 95%CI: −0.86, −0.21; *Z* = 3.20, *p* = 0.001). Heterogeneity was clearly significant for the pooled result of PPBG (*df* = 5, *p* < 0.0001, *I*^2^ = 76%). We thus carried out sensitivity analyses to investigate the potential sources of heterogeneity.

### Secondary Outcomes

#### Effect of Yoga on TC

**Figure 7:**
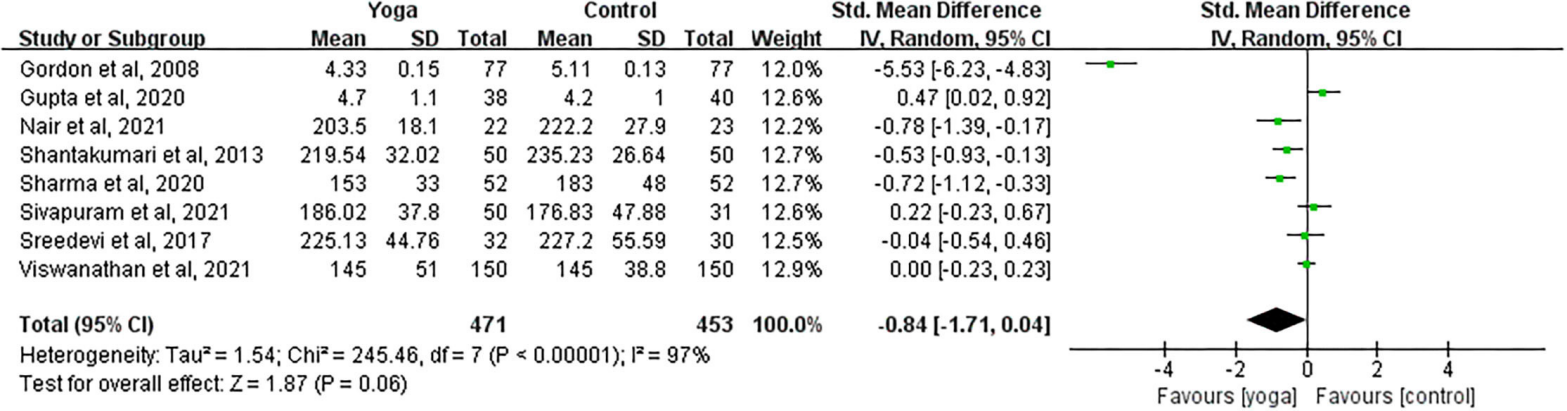
Forest plots of the effect of yoga on TC.

Data involving 924 subjects with T2DM from eight eligible studies were analyzed to find out the effect of yoga on total cholesterol (TC). Only four studies showed significant TC changes in the yoga groups following the intervention compared to the control groups (Figure 7). Results from the meta-analysis showed no significant overall standardized mean difference (SMD = −0.84; 95%CI: −1.71, 0.04; *Z* = 1.87, *p* = 0.06). Heterogeneity was clearly significant for the pooled result of TC (*df* = 7, *p* < 0.001, *I*^2^ = 97%).

#### Effect of Yoga on TG

**Figure 8:**
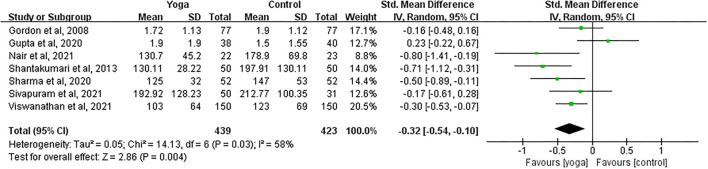
Forest plots of the effect of yoga on TG.

Data involving 862 subjects with T2DM from seven eligible studies were analyzed to determine the effect of yoga on triglycerides (TG). A total of four studies showed significant TG decreases in the yoga group following the intervention compared to the control group (Figure 8). Results from the meta-analysis showed a significant standardized mean difference favoring yoga group (SMD = −0.32; 95%CI: −0.54, −0.10; *Z* = 2.86, *p* = 0.004). Heterogeneity was clearly significant for the pooled result of TG (*df* = 6, *p* = 0.03, *I*^2^ = 58%). We carried out subgroup analyses to investigate the potential sources of heterogeneity.

#### Effect of Yoga on BMI

**Figure 9:**
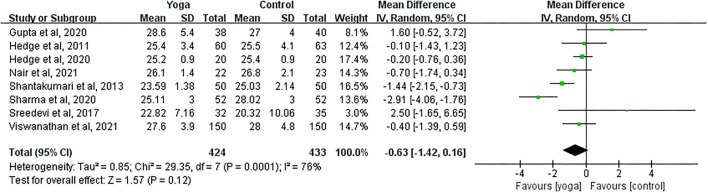
Forest plots of the effect of yoga on BMI.

Data involving 857 subjects with T2DM from eight eligible studies were analyzed to examine the effect of yoga on body mass index (BMI). The majority of the studies showed no significant BMI changes in the yoga groups following the intervention compared to the control groups (Figure 9). Results from the meta-analysis showed no significant overall mean difference between groups (MD = −0.63; 95%CI: −1.42, 0.16; *Z* = 1.57, *p* = 0.12). Heterogeneity was clearly significant for the pooled result of BMI (*df* = 7, *p* < 0.001, *I*^2^ = 76%).

### Heterogeneous Interpretation

#### HbA1c

**Figure 10:**
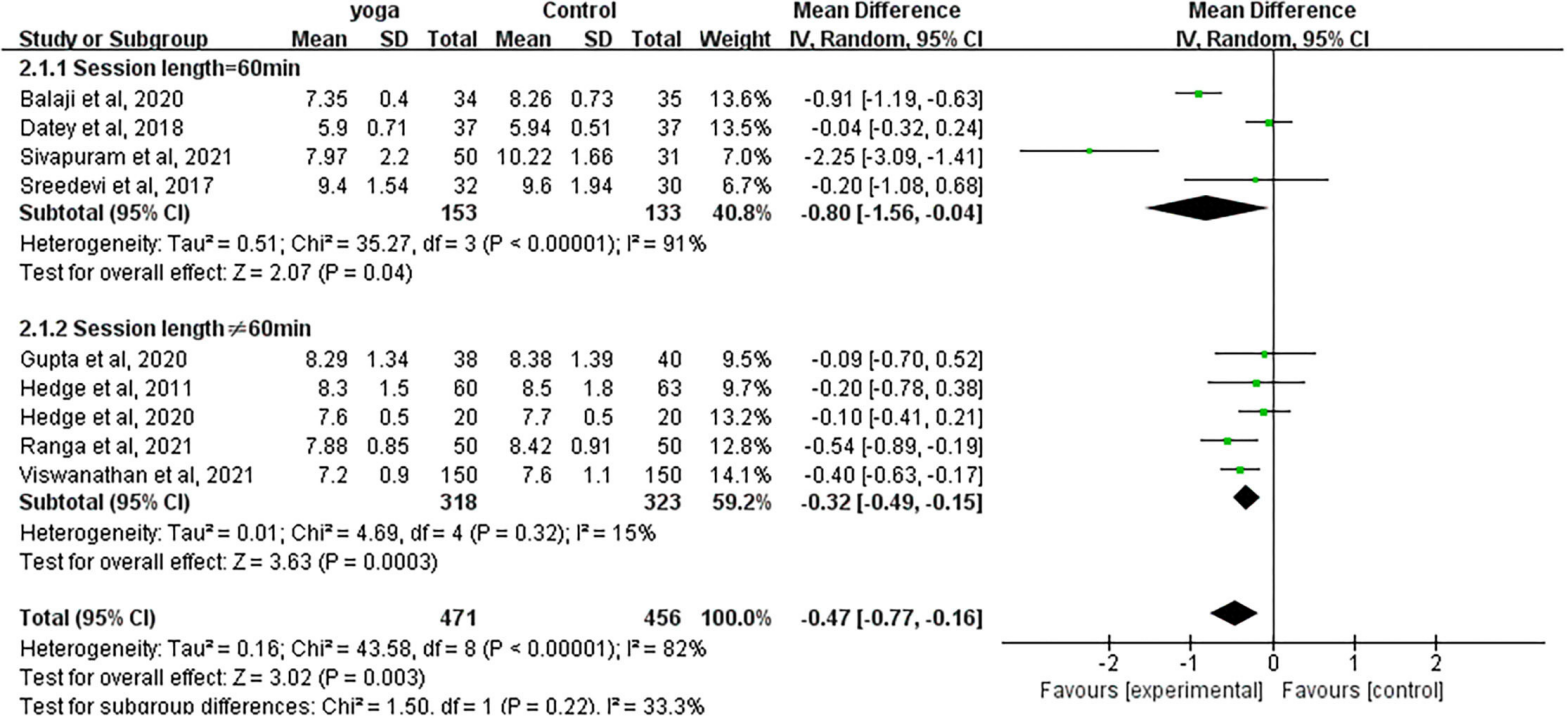
Forest plots of the subgroup analysis on Hba1c.

With subgroup moderator analysis, we observed that the effect of yoga differs according to the yoga session length. Subgroup analysis showed that (Figure 10) the heterogeneity was 15% when the session length was more or <60 min (MD = −0.32; 95%CI: −0.49, −0.15; *Z* = 3.63, *p* < 0.001); the heterogeneity was 91% when each session was equal to 60 min (MD = −0.80; 95%CI: −1.56, −0.04; *Z* = 2.07, *p* = 0.04). The heterogeneity increased after pooling the data, indicating that the difference in each session length was likely to be the source of heterogeneity.

#### FBG

**Figure 11:**
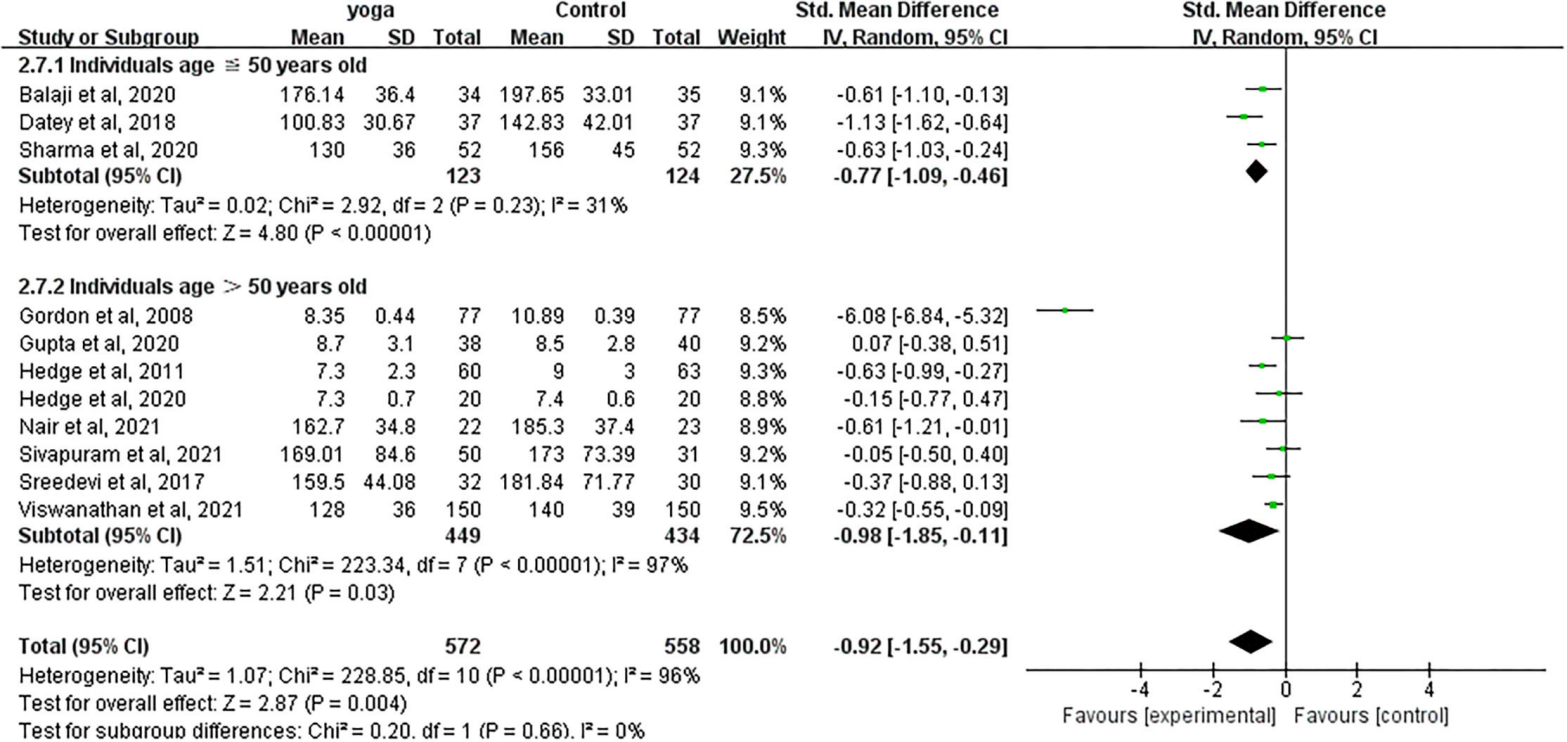
Forest plots of the subgroup analysis on FBG.

With subgroup moderator analysis, we observed that the effect of yoga on FBG differs according to the individual's age. Subgroup analysis result showed (Figure 11) that patients ≤50 years old were with 31% heterogeneity (SMD = −0.77; 95%CI: −1.09, −0.46; *Z* = 4.80, *p* < 0.001); patients older than 50 years old were with 97% heterogeneity (SMD = −0.98; 95%CI: −1.85, −0.11; *Z* = 2.21, *p* = 0.03). The heterogeneity increased after pooling the data, indicating that difference in each individual's age was likely to be the source of heterogeneity.

#### PPBG

With subgroup moderator analysis, we did not observe that the effect of yoga differs according to how they were grouped. Further, a sensitivity analysis identified that a more restricted analysis of the data did not affect the merging effect size. After excluding the study with high heterogeneity (Balaji et al., 2020), the pooled data of the rest studies changed insignificantly, indicating that the sensitivity was low, and the results were relatively robust.

#### TG

**Figure 12:**
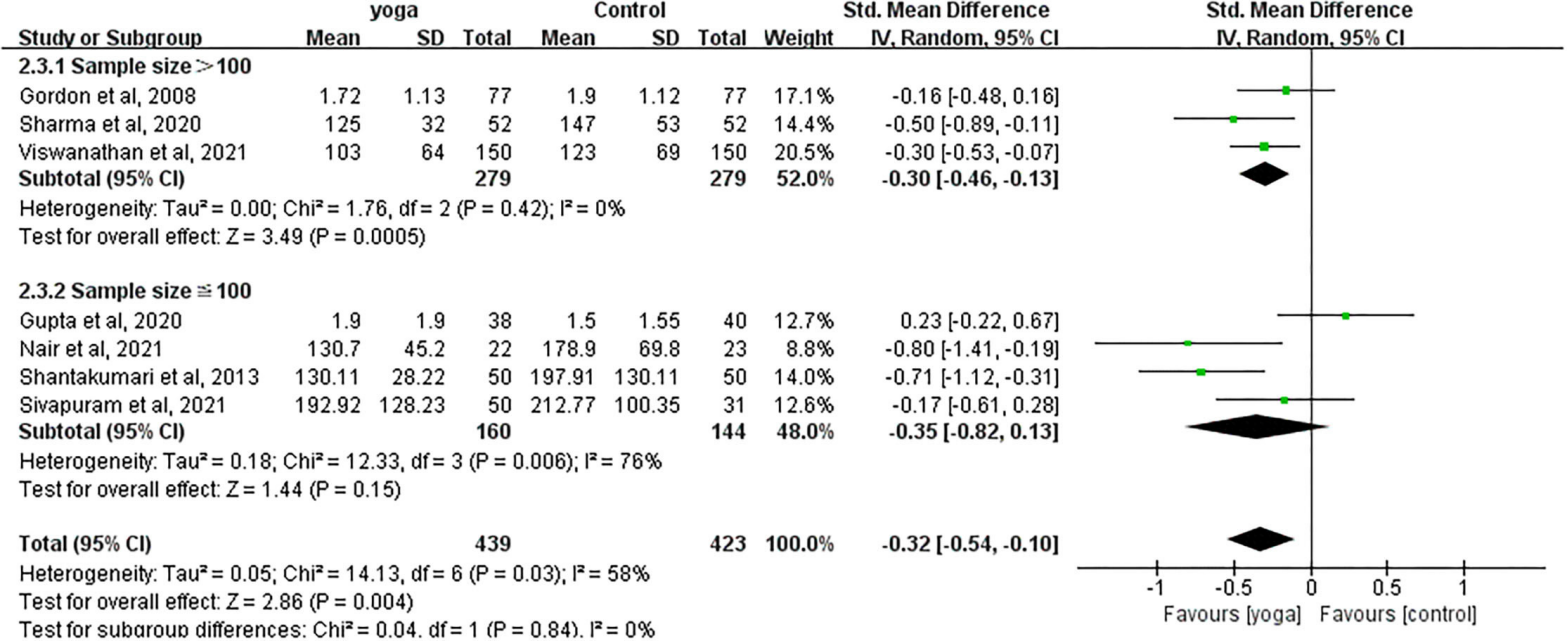
Forest plots of the subgroup analysis on TG.

With subgroup moderator analysis, we observed that the effect of yoga differs according to the sample size of the study. Subgroup analysis showed that (Figure 12) the heterogeneity was 0% when the sample size outweighed 100 subjects (SMD = −0.30; 95%CI: −0.46, −0.13; *Z* = 3.49, *p* < 0.001); the heterogeneity was 76% when sample size was ≤100 subjects (SMD = −0.35; 95%CI: −0.82, 0.13; *Z* = 1.44, *p* = 0.15). The heterogeneity increased after pooling the data, indicating that difference in each sample size was likely to be the source of heterogeneity.

#### Publication Bias

**Figure 13:**
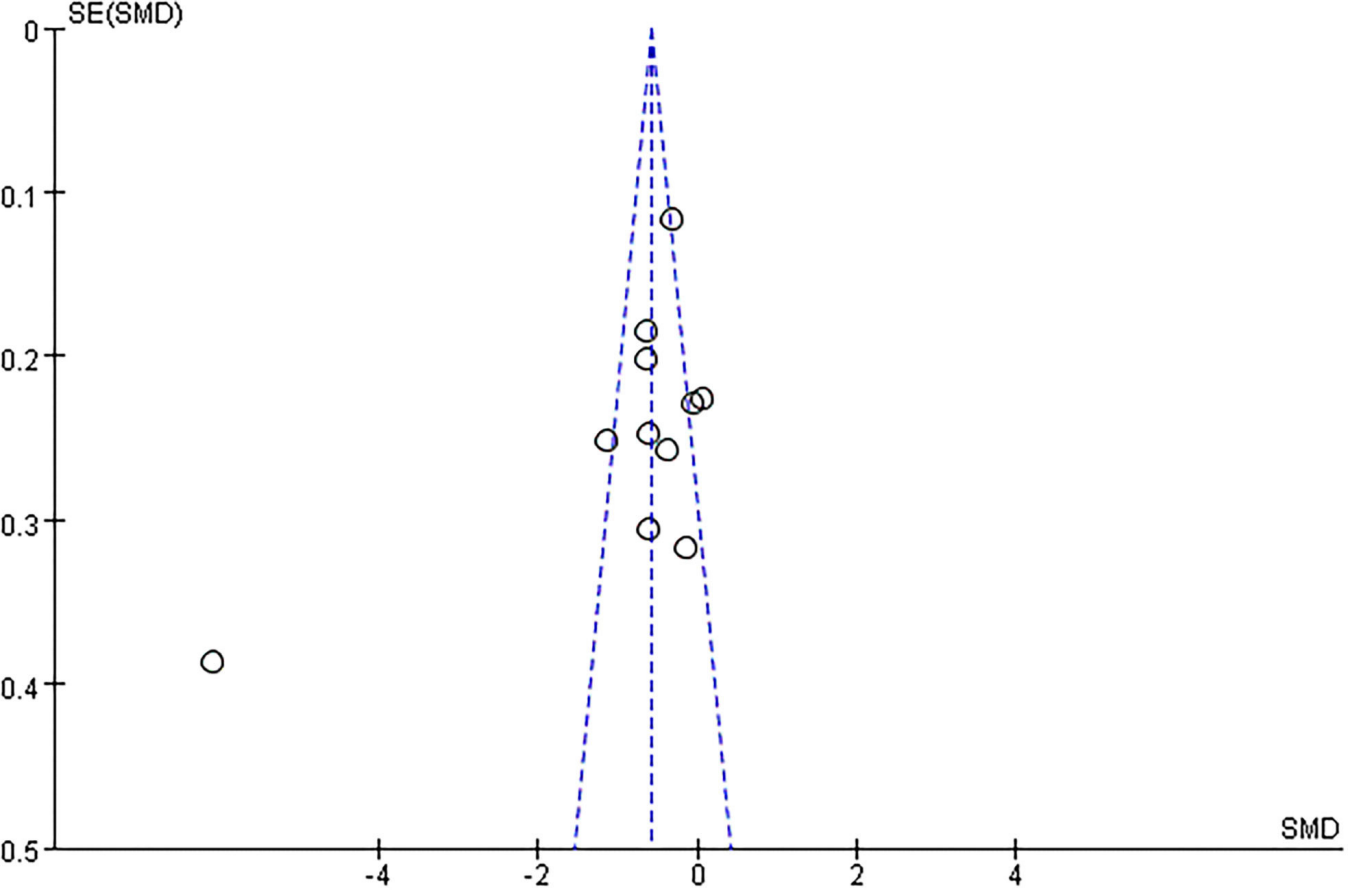
Funnel plot on FBG of patients with T2DM.

Only when the number of studies involving in the meta-analysis was more than ten, a funnel plot was eligible for assessing publication bias. The funnel plots for outcomes of FBG looked approximately asymmetrical as assessed by visual examination, indicating a high possibility of bias (Figure 13).

## Discussion

The purpose of the study was to evaluate available evidence from existing RCTs concerning the effect of yoga-based intervention on the biochemical indicators of blood glucose and lipid profile in patients with T2DM. Chronic hyperglycemia was the major characteristic of T2DM, which was commonly diagnosed depending on HbA1c, FBG, and PPBG levels (American Diabetes Association, 2016). The results of the meta-analyses incorporating evidence from the 13 RCTs with a total of 1,335 patients with T2DM suggested that yoga can significantly improve HbA1c, FBG, PPBG, and TG levels. The study supported that yoga training improves blood glucose control and prevents T2DM biomarkers from worsening. The synthesized evidence approved yoga as an effective complementary treatment for patients with T2DM. These findings were in line with a recently published meta-analysis yielding favorable effects of yoga on specific metabolic syndrome (Chu et al., 2016).

Although not a primary focus of this study, an insignificant effect of yoga practice on BMI was observed, which is consistent with prior work (Boulé et al., 2001). A possible reason behind this was that yoga training decreases muscle insulin resistance and increases glucose disposal through a number of mechanisms that would not necessarily be relevant to losing weight. However, results must be interpreted with caution because of the limited number of selected studies in the analysis. Therefore, additional investigation is necessary to discover the impact of yoga practice on body mass.

Furthermore, there was significant heterogeneity among primary and secondary outcome variables across different studies, which remained even after adjusting the impacts of different types of intervention and characteristics of participants (potential moderators). This indicated that there were still important variations between the included studies that made them considerably different from each other. We explained all results except for PPBG through subgroup analysis. For the pooled result of PPBG, after excluding the study with high heterogeneity (Balaji et al., 2020), the pooled data from the rest studies showed no substantial change, indicating that the sensitivity was low, and the results were relatively reliable.

Another aspect that needs to be taken into account while explaining these results was that in cases where diabetes treatment adopting both pharmaceutical and yoga approaches yield sizable effect, yoga as an ingredient to such treatment was hard to ascertain its sole effectiveness. All studies included in the present meta-analyses administered yoga to the participants together with other pharmaceutical treatments. Given such a condition, caution should be taken when concluding that the favorable effect on T2DM was solely due to the adjunct yoga treatment. Because both treatment and control groups incorporated pharmacological intervention, these favorable effects may also be caused either by positive yoga–medicine interaction or yoga alone. To observe or repeat these add-on effects, yoga is better recommended as an adjuvant treatment to pharmaceutical prescriptions for patients with diabetes.

Even though RCT is widely believed to provide the most reliable evidence of causality for clinical tests, the RCTs selected for these meta-analyses had extra problems through the lens of risk of bias. The overall risk of bias for each included trial was either unclear or high. Typically, only statistically significant results suggesting a favorable effect are more likely to be published. Moreover, although yoga has been reported to be a generally safe exercise (McCall et al., 2013), in the context of T2DM, none of the studies reported any adverse events following a yoga program. It is not known whether in other studies, participants experienced any adverse events, or whether authors failed to report these adverse events. Finally, it is a strong recommendation from this study that more RCT-based examinations should be carried out revealing how yoga as an adjuvant therapy plays its role in T2DM treatment. Additionally, it may need a longer time for yoga to take its effect on diabetes, and thus, future research should incorporate interventions with a longer intervening session to monitor the changes in blood glucose, blood lipids, and body composition.

This study depicted the influence of yoga as a complementary treatment on biochemical indicators of blood glucose and lipid profile in patients with T2DM. The main limitation of this review is information insufficiency from the included studies, such as some studies did not report age and session length, which could have an impact on the results. Therefore, caution should be seriously taken concluding the reductive effect of yoga based on the extracted information. It might be possible that the overall effect sizes change substantially if the data reports were more complete. Publication bias and small sample sizes also did not make us convincingly state that the short-term effects of yoga interventions can be generalized to the long-term management of T2DM. Whereas, we strictly controlled the quality of the included studies, the fact is that the pooled results of the meta-analyses still showed large heterogeneity despite our attempts to identify possible sources of heterogeneity through subgroup analysis. Additionally, due to our limited access to online databases, our search may miss some databases such as Scopus. Finally, as indicated in the results, the majority of RCTs published to date exploring T2DM's response to yoga originated in India, suggesting the need for conducting strict trials with patients with T2DM of other races and/or in other developed and developing countries.

## Conclusions

The findings of this study suggested that yoga treatment can improve the indices of blood glucose and lipid profile in patients with T2DM (simultaneously receiving pharmacological treatment). Therefore, yoga can be regarded as an effective complementary treatment to T2DM for the short term (i.e., 10–24 weeks). Future research is needed to highlight high-quality trials with standardized yoga plans to verify the long-term reductive effect of yoga on T2DM-related indicators. However, given the aforementioned limitations and potential bias in the present study, more large-scale and rigorous RCTs must be carried out to reaffirm our current findings.

## Data Availability Statement

The original contributions presented in the study are included in the article/Supplementary Material, further inquiries can be directed to the corresponding author/s.

## Author Contributions

SC participated in the search strategy, data analysis, heterogeneous interpretation, and manuscript writing. SC, SD, and YL participated in the data extraction. SD and YL reviewed the search results and critical review of the manuscript. TY participated in the preliminary study design. All authors contributed to the article and approved the submitted version.

## Funding

This study was supported by the Fundamental Research Funds for the Central Universities.

## Conflict of Interest

The authors declare that the research was conducted in the absence of any commercial or financial relationships that could be construed as a potential conflict of interest.

## Publisher's Note

All claims expressed in this article are solely those of the authors and do not necessarily represent those of their affiliated organizations, or those of the publisher, the editors and the reviewers. Any product that may be evaluated in this article, or claim that may be made by its manufacturer, is not guaranteed or endorsed by the publisher.
